# Evaluation of the C-Terminal Fragment of *Entamoeba histolytica* Gal/GalNAc Lectin Intermediate Subunit as a Vaccine Candidate against Amebic Liver Abscess

**DOI:** 10.1371/journal.pntd.0004419

**Published:** 2016-01-29

**Authors:** Xiangyang Min, Meng Feng, Yue Guan, Suqin Man, Yongfeng Fu, Xunjia Cheng, Hiroshi Tachibana

**Affiliations:** 1 Department of Medical Microbiology and Parasitology, School of Basic Medical Sciences, Fudan University, Shanghai, China; 2 Department of Infectious Diseases, Tokai University School of Medicine, Isehara, Kanagawa, Japan; Jawaharlal Nehru University, INDIA

## Abstract

**Background:**

*Entamoeba histolytica* is an intestinal protozoan parasite that causes amoebiasis, including amebic dysentery and liver abscesses. *E*. *histolytica* invades host tissues by adhering onto cells and phagocytosing them depending on the adaptation and expression of pathogenic factors, including Gal/GalNAc lectin. We have previously reported that *E*. *histolytica* possesses multiple CXXC sequence motifs, with the intermediate subunit of Gal/GalNAc lectin (i.e., Igl) as a key factor affecting the amoeba's pathogenicity. The present work showed the effect of immunization with recombinant Igl on amebic liver abscess formation and the corresponding immunological properties.

**Methodology/Principal Findings:**

A prokaryotic expression system was used to prepare the full-length Igl and the N-terminal, middle, and C-terminal fragments (C-Igl) of Igl. Vaccine efficacy was assessed by challenging hamsters with an intrahepatic injection of *E*. *histolytica* trophozoites. Hamsters intramuscularly immunized with full-length Igl and C-Igl were found to be 92% and 96% immune to liver abscess formation, respectively. Immune-response evaluation revealed that C-Igl can generate significant humoral immune responses, with high levels of antibodies in sera from immunized hamsters inhibiting 80% of trophozoites adherence to mammalian cells and inducing 80% more complement-mediated lysis of trophozoites compared with the control. C-Igl was further assessed for its cellular response by cytokine-gene qPCR analysis. The productions of IL-4 (8.4-fold) and IL-10 (2-fold) in the spleen cells of immunized hamsters were enhanced after *in vitro* stimulation. IL-4 expression was also supported by increased programmed cell death 1 ligand 1 gene.

**Conclusions/Significance:**

Immunobiochemical characterization strongly suggests the potential of recombinant Igl, especially the C-terminal fragment, as a vaccine candidate against amoebiasis. Moreover, protection through Th2-cell participation enabled effective humoral immunity against amebic liver abscesses.

## Author Summary

Amebiasis, a neglected tropical disease caused by the protozoan parasite *Entamoeba histolytica*, is the second leading cause of death from protozoan diseases. Vaccination is considered as an effective strategy against amebiasis; however, clinical vaccines have yet to be developed. We previously reported that the intermediate subunit of Gal/GalNAc lectin (Igl) of *E*. *histolytica* is a key factor related to the adherence and cytotoxicity of this parasite to host cells. This study focused on the immune efficacy and immunological characterization of recombinant Igl and its fragments. Highly effective protection was observed in the hamsters immunized intramuscularly with the C-terminal fragment of Igl (C-Igl). C-Igl was further assessed to determine the immunological basis of protection. The immunized hamsters generated high levels of specific antibodies; these hamsters also showed an enhanced complementary-mediated lysis. The spleen cells from the immunized hamsters produced the cytokines IL-4, IL-10, and programmed cell death 1 ligand 1 after these cells were stimulated by C-Igl *in vitro*. These results demonstrate that recombinant Igl, particularly the C-terminal fragment, is a candidate vaccine for amebiasis. Nevertheless, further studies on Igl should be conducted to explore the preliminary steps of the development of vaccines for human amebiasis.

## Introduction

Amebiasis caused by infection with *Entamoeba histolytica* is one of the most problematic parasitic diseases affecting people in both developing and developed countries. *E*. *histolytica* causes an estimated 50 million collective cases of dysentery, colitis, and extraintestinal abscesses resulting in 40,000–100,000 deaths annually [1–4]. Its mortality is ranked third among parasitic diseases. Despite the medical importance of this parasite, an effective vaccine to prevent amebiasis has yet to become available. However, several *E*. *histolytica* antigens have been proposed as potential vaccines, including galactose- and *N*-acetyl-D-galactosamine (Gal/GalNAc)-inhibitable lectin [5–11], serine-rich protein [12, 13], peroxiredoxin [14], lipophosphoglycan [15, 16], and metallosurface protease [17, 18]. The molecule showing the highest potential among these candidates is the 170 kDa heavy subunit of Gal/GalNAc-inhibitable lectin (Hgl) [19–21]. In particularly the LecA domain (578 aa to1154 aa) in Hgl is considered to be a potential vaccine [8].

A glycosylphosphatidylinositol-anchored 150 kDa intermediate subunit (Igl) is non-covalently associated with Hgl and contributes to adherence [22–24]. Immunization with native Igl as well as passive immunization with a mouse monoclonal antibody (mAb) to Igl can inhibit amebic liver abscess (ALA) formation in hamsters [25, 26]. The two isoforms of Igl, namely, Igl-1 and Igl-2, are cysteine-rich proteins containing multiple CXXC motifs [27]. Igl was identified as a lectin because it binds to a Gal-affinity column. *E*. *histolytica* Igl has also been detected in addition to Hgl in the protein fraction that binds to GalNAc-bovine serum albumin-coated magnetic beads [28]. However, the amino acid sequences of both Igls lack a known carbohydrate recognition domain of other lectins. Igl-1 seems to be more closely associated with the pathogenicity of *E*. *histolytica* [29]. Igl-1 is a major target of the humoral immune response in seropositive individuals. Upon evaluating the reactivity of sera from patients with amebiasis to various Igl-1 fragments, namely, the N-terminal (N-Igl), middle (M-Igl), and C-terminal (C-Igl) fragments, the highest sensitivity was observed in C-Igl [30]. All sera from asymptomatic patients reacted with M-Igl and C-Igl, suggesting that antibodies to the epitopes located in M-Igl and C-Igl function in preventing the invasion of trophozoites into the host tissue. Human mAb recognizing N-Igl does not inhibit amebic adherence to Chinese hamster ovary (CHO) cells, whereas another mAb that is reactive with M-Igl and C-Igl inhibited adherence [31].

In this study, we evaluate Igl-1 (rIgl-1) and three fragments, N-Igl, M-Igl, and C-Igl, as potential recombinant vaccines in hamster model. We also analyze the significance of humoral, cell-mediated immune responses and programmed cell death 1 (PD1) signaling pathway in protection from ALA.

## Methods

### Ethics statement

All animal experiments were performed in strict accordance with guidelines from the Regulations for the Administration of Affairs Concerning Experimental Animals (1988.11.1) and were approved by the Institutional Animal Care and Use Committee (IACUC) of our institutions (Permit Number: 054001, 065001, and 20110307–051). All efforts were made to minimize suffering.

### Animals and ameba

Groups of three- to four-week-old male hamsters were obtained from Japan SLC, Inc. and Shanghai Songlian Experimental Animal Factory for vaccination. Male BABL/c mice, aged four to six weeks, were purchased from Shanghai SLAC Laboratory Animal Co., Ltd. for experiments on Igl-elicited cytokines. Trophozoites of *E*. *histolytica* SAW755CR and HM-1:IMSS strains were grown under axenic conditions at 36.5°C in YIMDHA-S medium [32] containing 15% (v⁄v) heat-inactivated adult bovine serum. Parasites were grown for 72 h (log phase) for use in all experiments.

### Preparation of vaccine antigens

The recombinant *E*. *histolytica* antigens (rIgl-1, rIgl-2, N-Igl, M-Igl, and C-Igl) were prepared as previously described [30]. Briefly, the target plasmids were transformed in host bacteria *Escherichia coli* BL21 Star (DE3) pLysS and inclusion bodies were harvested after inducement. The refolding of proteins were conducted using the Protein Refolding Kit (Novagen) and then refolded proteins were further purified using a Ni-NTA His•Bind Resins kit (Novagen) in accordance with the manufacturer’s instructions. The purity of the recombinant antigens was analyzed by sodium dodecyl sulfate-polyacrylamide gel under reducing conditions. After preparing recombinant antigens, approximately 50 μg of each recombinant protein was intraperitoneally injected into the BABL/c mice to test the toxicity of these proteins. Contaminating residual endotoxin was quantified using a commercial kit (EKT-5M, Jin Shan Chuan, Co., Ltd., China). All animals received less than 4.8 EU/kg per dose of antigen in accordance with the accepted guidelines for preclinical research.

### Immunization course

A total of 15 heads of 40 g to 50 g hamsters were intramuscularly immunized with 50 μg of rIgl-1 emulsified in TiterMax Gold (Sigma–Aldrich) adjuvant. Boosters were inoculated on days 21 and 35 with the same amount of rIgl-1 in incomplete Freund’s adjuvant. The control group comprising 12 heads of hamsters was sham-immunized with PBS in adjuvant. Before the challenge, a small amount of serum from each hamster was isolated for the serological test and adherence inhibition assay. In addition, 24 heads of 40 g to 50 g hamsters were intramuscularly immunized with 50 μg of N-Igl, M-Igl, and C-Igl emulsified in TiterMax Gold adjuvant in an identical course described above. The control group, which included eight hamsters, was immunized with PBS in adjuvant. Thirdly, 15 heads of 40 g to 50 g hamsters were intramuscularly immunized with 50 μg of C-Igl emulsified in TiterMax Gold adjuvant in an identical course described above. The control group, which included 12 hamsters, was immunized with PBS in adjuvant.

To assess antigen-specific cell-mediated immune response, three vaccination experiments were conducted by using 40 heads of 40 g to 50 g hamsters intramuscularly immunized with C-Igl (10, 10 and 20 hamsters each in three experiments) and 20 heads of 40 g to 50 g hamsters treated as control (5, 5 and 10 hamsters each in three experiments). The immune courses were identical to those described above.

### Animal model for ALA

Two weeks after the last immunization, ALA was induced by direct inoculation of 1 × 10^6^ axenic *E*. *histolytica* SAW755CR trophozoites into the liver as previously described [26]. The hamsters were sacrificed at day seven post-infection. The percentages of liver abscess were calculated as the weight of the abscess divided by the liver weight recorded prior to abscess removal. Vaccine efficacy was calculated as [(rate of unvaccinated animals infected-rate of vaccinated animals infected) / (rate of unvaccinated animals infected)] × 100.

### Measurement of immuno-reactivity by ELISA, western immunoblot and dot blot analysis

To confirm antigen-specific antibody responses, 40 sera from C-Igl-immunized hamsters and 20 sera from sham-immunized hamsters were used. The antigen-specific binding of IgG to C-Igl, rIgl-1, and rIgl-2 were analyzed by ELISA as previously described [31]. Briefly, ELISA plates were coated with C-Igl, rIgl-1, or rIgl-2 and then treated with PBS containing 1% skim milk. Sera from immune or sham immune hamsters diluted 1:4,000, 1:10,000, 1:25,000, or 1:62,500 with PBS were added to the wells. After washing, the wells were incubated with horseradish peroxidase (HRP) conjugated goat antibody to hamster IgG (MP Biomedicals, LLC), after developing the OD490 was recorded. C-Igl specific IgG isotypes (IgG1 and IgG2) response were determined by using a mice IgG subclass ELISA kit (Bio-rad, USA).

Aliquots of total protein extracts from *E*. *histolytica* HM-1:IMSS strain were separated through sodium dodecyl sulphate–polyacrylamide gel electrophoresis under reduced condition. The proteins were transferred from the gel onto a polyvinylidene difluoride membrane. The protein blots were blocked with 3% skim milk in PBS and then incubated with pooled sera from C-Igl- or sham-immunized hamsters. The blots were then incubated with HRP-conjugated goat anti-hamster IgG. After each incubation step, the membrane was washed with PBS containing 0.05% Tween 20. Proteins were detected using an enhanced HRP-DAB substrate detection kit (Tiangen Biotech, China).

For the dot blotting experiment, rIgl-1 and rIgl-2 were blotted on the nitrocellulose membrane as described [29]. Filter strips were blocked with 5% bovine serum albumin in PBS and reacted with diluted pooled sera from C-Igl- or sham-immunized hamsters for 30 min. HRP-labeled goat anti-hamster IgG antibody (MP Biomedicals) was used as second antibody. The strips were developed with an Enhanced HRP-DAB Chromogenic Substrate Kit (Tiangen Biotech, China).

### CHO cell adherence assay

CHO cells were grown and maintained in F12 medium, whereas the *E*. *histolytica* strain HM-1: IMSS trophozoites were grown in YIMDHA-S medium containing 15% (v⁄v) heat-inactivated adult bovine serum. The adherence assay, using 15 sera from rIgl-1-immunized, 8 sera from C-Igl-immunized, or 20 sera from sham-immunized hamsters, was conducted as previously described [26, 33]. In brief, *E*. *histolytica* trophozoites were preincubated with 1:10, 1:100, and 1:1,000 diluted pooled hamster sera. The treated trophozoites were incubated with CHO cells on ice for 2 h, then the number of trophozoites with at least three adherent CHO cells was scored by examining at least 300 trophozoites. Adherence was expressed as the percentage of adherence seen in PBS-treated controls. The experiments were repeated three times.

### Assay of cytolytic effect on ameba by complement

The assay system to determine the activation of the classical complement cascade was performed using a previously reported protocol [18, 34, 35] with modification, 40 sera from C-Igl-immunized hamsters and 20 sera from sham-immunized hamsters were used. Guinea pigs’ complement (Cedarlane, Canada) was used as the source of complement. *E*. *histolytica* trophozoites were harvested and washed with cold PBS three times, and the final concentration was adjusted to 2 × 10^6^/ml in PBS containing 0.15 mM CaCl_2_ and 0.5mM MgCl_2_. Pretreatment of samples for 30 min with EGTA was used to block the classical complement pathway. A 25 μl portion of trophozoites was mixed with different concentrations (1:10, 1:100, and 1:1,000) of pooled sera from C-Igl-immunized or sham-immunized or normal hamsters. An equal volume of different concentrations (1:20, 1:40, and 1:80) fresh or heat-inactivated (30 min, 56°C) guinea pigs’ complement was added and then incubated for 1 h at 37°C. Lysis was determined by microscopic analysis after staining with trypan blue, preparations were always conducted in quadruplicate. The experiments were repeated four times.

### Analysis of cytokine gene expression by one-step reverse transcription PCR

Spleen cells from sham- and C-Igl-immunized hamsters were harvested after 2 weeks of the last immunization and the spleen cells (2 × 10^6^) were cultured at 37°C in 5% CO_2_ in 24-well culture plates (Costar) containing RPMI 1640 medium (Invitrogen) supplemented with 10% fetal bovine serum, 100 U/ml penicillin, and 100 μg/ml streptomycin. The cells were stimulated with 5 μg/ml C-Igl protein for the antigen-specific cell-mediated immune response. After incubation for 48 h, all cells were collected. Total RNA of the spleen cells from the sham-immunized or C-Igl-immunized hamsters was purified with an RNeasy Plus Mini kit (Qiagen, USA). One-step reverse transcription-PCR (one-step RT-PCR) was performed using the PrimeScript™ One Step RT-PCR Kit (TAKARA). The first-strand cDNA synthesis reaction and PCR reaction were performed in the same tube. Gene-specific primers for IL-4, IL-6, IL-10, INF-γ, and TNF-α were utilized for one-step RT-PCR and β-actin transcripts were used as an internal reference control (Table 1) [36, 37]. Positive and negative controls were stimulated with concanavalin A (ConA) and PBS, respectively. The one-step RT-PCR products were analyzed using 2% agarose gels stained with 2 mg/ml ethidium bromide to monitor the presence of the expected size bands. Each experiment was performed at least three times.

**Table 1:** Primers used in the current study.

Cytokine		Accession number	Sequence (From 5′ to 3′)	Amplicon size (bp)	Annealing Temperature	Elongation time (sec)
Hamster-IFNγ	S:	AF034482	CCATCCAGAGGAGCATAG	211	60	15
	AS:		CAGCACCGACTTCTTTTC			
Hamster-TNFα	S:	AF046215	CCTCCTGTCCGCCATCAAG	125	60	15
	AS:		CACTGAGTCGGTCACCTTTC			
Hamster-IL4	S:	AF046213	TGCACCGAGATGGTCGTAC	153	60	15
	AS:		GTCTTTGAGAACCCTGGAAT			
Hamster-IL6	S:	AB028635	AGGACACTACTCCCAACAG	194	58	15
	AS:		GAGGCATCCATCATTTATT			
Hamster-IL10	S:	Reference 40	CAACTGCAGCGCTGTCATCGATTT	110	60	15
	AS:		ATGGCCTTGAAGACGCCTTTCTCT			
Hamster-β-actin	S:	Reference 41	TCTACAACGAGCTGCG	357	60	30
	AS:		CAATTTCCCTCTCGGC			
Hamster-PD1	S:	XM005077183	GTTTCAAGGCGTGGTCATT	195	-	-
	AS:		AAGTCCAGCTCCTCATAGGC			
Hamster-PD1-L1	S:	XM005063709	GCGGGTGTTTACGGTTGT	126	-	-
	AS:		ATGCTCGGACGTGACTGG			
Hamster-PD1-L2	S:	XM005063214	GGCAGTACCGCTGTCTGGT	210	-	-
	AS:		TGATGTGGCTGGTGTTGG			

### Determination of immune-associated gene expression by real-time PCR

Total RNA (1 μg) of the spleen cells from the sham-immunized or C-Igl-immunized hamsters was purified with an RNeasy Plus Mini kit (Qiagen, USA). The cDNA was synthesized with a Primescript first-strand cDNA synthesis kit (Takara, Japan) using oligo(dT) primers. Quantitative real-time PCR (qRT-PCR) was carried out in a final reaction volume of 20 μl according to the manufacturer’s recommendations on an ABI 7500 Real-time PCR system (Applied Biosystems, USA). Reactions were performed in a 96-well plate with SYBR Premix Ex Taq (Takara, Japan), which contained primers for each cytokine gene and PD1 and PD1 ligands (PD1-L1 and PD1-L2), with β-actin as the reference gene (Table 1). The amplification cycling conditions were as follows: 30 s at 95°C and 40 cycles of 5 s at 95°C and 35 s at 60°C. Analysis by qRT-PCR of gene expression for each cytokine was conducted during the log phase of product accumulation during which the Ct values correlated linearly with relative DNA copy numbers. Each experiment was performed at least three times.

### Statistical analysis

All statistical analyses were performed using IBM SPSS (version 20, SPSS Statistics/IBM Corp., Chicago, IL, USA). Hamster protection data were analyzed using a chi-square test (Fisher’s exact probability test) and CHO cell adherence experiments, complement lysis assay, and ELISA data were analyzed using the two-tailed Student’s t-test. The analysis of qRT–PCR data was performed using the two-tailed Mann–Whitney U test. *P* < 0.05 was considered statistically significant.

## Results

### Effects of vaccination with rIgl-1 and three Igl fragments on ALA formation

In the first line of experiments, rIgl-1 was used to immunize the hamsters by intramuscular injection. After immunization, the level of specific antibodies to the antigen was evaluated. The results of indirect fluorescent antibody test (IFA) and ELISA confirmed that none of the controls but all of the animals immunized with rIgl-1 developed positive serum IgG antibodies, the median endpoint titer of rIgl-1-immunized sera to *E*. *histolytica* trophozoites was 1:2,560 in IFA and the endpoint titers of rIgl-1-immunized sera to the recombinant protein were more than 1:25,600 in ELISA. Seven days after the challenge, in the sham-immunized group, ALA formation was observed in 10 out of 12 hamsters. The mean percentage of liver abscessed in the 10 hamsters was 30.5%. By contrast, ALA was formed in only one of 15 hamsters immunized with rIgl-1. Even in the single non-protected hamster, the abscess size was 3% in the liver. (Table 2).

**Table 2:** Protection of hamsters from amebic liver abscess formation by vaccination with rIgl-1 and three Igl fragments.

Group	Vaccination	No. of hamsters with abscesses/No. challenged	Vaccine efficacy (%)	% of liver abscessed in non-protected hamsters (mean ± SD)
Experiment 1				
Control	PBS	10/12		30.5±17.3
1	rIgl-1	1/15^a^	92	3
Experiment 2				
Control	PBS	8/8		27±21
1	N-Igl	8/8	0	26±16.2
2	M-Igl	6/8	25	25±20.7
3	C-Igl	0/8^b^	100	-
Experiment 3				
Control	PBS	12/12		23.9±5.2
1	C-Igl	1/15^c^	93	4

^a^ Two-tailed Fisher’s exact test. *P* < 0.001

^b^ Two-tailed Fisher’s exact test. *P* < 0.001

^c^ Two-tailed Fisher’s exact test. *P* < 0.001

In the second line of experiments, the effects of the three partial fragments of N-Igl, M-Igl, and C-Igl were investigated. In the control group immunized with PBS and adjuvant, ALA was observed in all eight hamsters. No protective effect was observed in the hamsters immunized with N-Igl, whereas two out of eight hamsters immunized with M-Igl did not show ALA formation. By contrast, complete protection from ALA formation was observed in the group immunized with C-Igl (vs. control, *P* < 0.001) (Table 2).

In the third line of experiments, the effects of C-Igl were investigated again. In the control group immunized with PBS and adjuvant, ALA was observed in all of 12 hamsters. ALA was formed in only one of 15 hamsters immunized with C-Igl (vs. control, *P* < 0.001). In the single non-protected hamster, the abscess size was 4% in the liver (Table 2).

### *In vitro* effect of immune sera on amebic adherence and complement-mediated lysis of trophozoites

**Fig 1:**
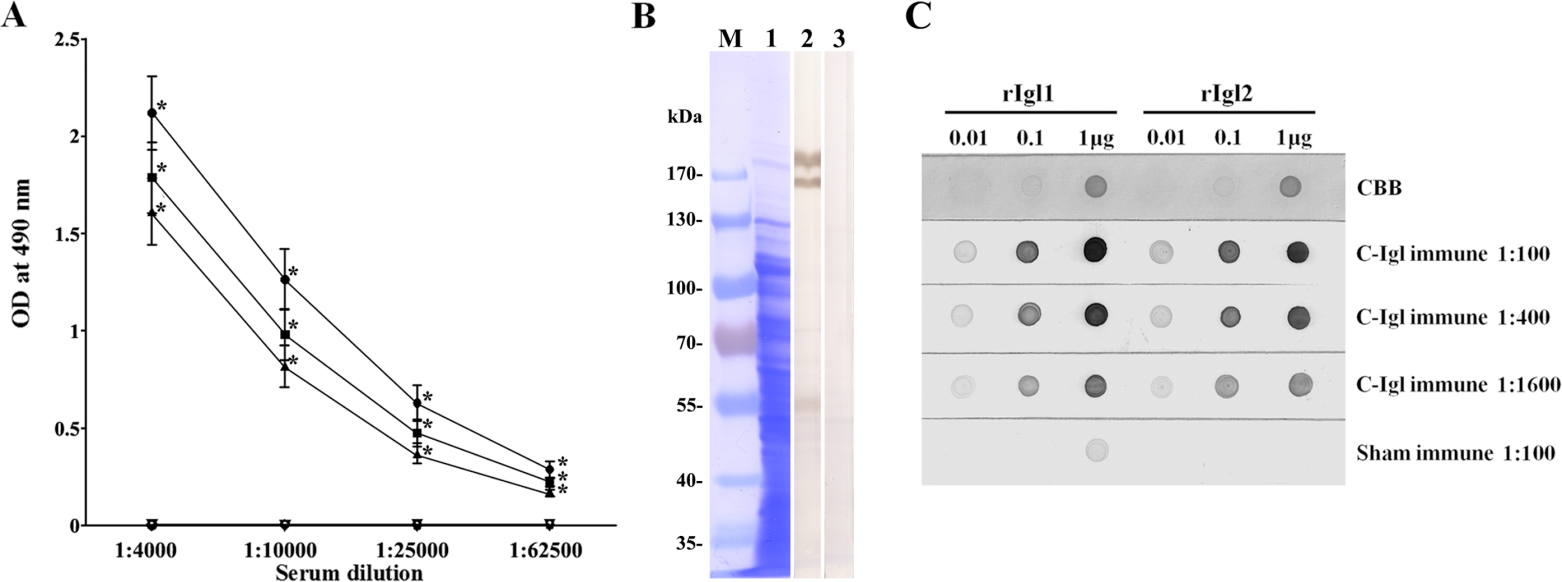
Reactivity of sera from hamsters immunized with C-Igl and sham-immunized hamsters to C-Igl, rIgl1, and rIgl2 in ELISA (A), Western immunoblot (B) and dot blot analysis (C). (A) Mean value of ELISA results from serial dilutions of sera from C-Igl-immunized hamsters reacted with C-Igl (●), rIgl-1 (■), or rIgl-2 (▲) and sera from sham-immunized hamsters reacted with C-Igl (**▽**), rIgl-1 (◇), or rIgl-2 (○). Error bars represent the standard errors of the means calculated from each individuals from three independent experiments.*P < 0.001. (B) Western immunoblot analysis of the reactivity of pooled sera from immunized hamsters to *E*. *histolytica* trophozoites. Crude antigen from the HM-1: IMSS strain was subjected to SDS-PAGE under reduced conditions. The strip in lane 1 was stained with CBB. Strips were treated with 1:400 diluted sera from C-Igl-immunized hamsters (lane 2) and sham-immunized hamsters (lane 3). Biostep Prestained Protein Marker (Tanon, China) was used and numbers to the left indicate the molecular masses of the marker. (C) Various concentrations (0.01, 0.1, and 1 μg) of rIgl-1 and rIgl-2 were spotted on nitrocellulose membranes. One strip was stained with Coomassie brilliant blue (CBB). Other strips were treated with different dilutions of pooled sera. HRP-conjugated goat antibody to hamster IgG was used as a secondary antibody.

The reactivity of C-Igl-immunized hamsters’ sera to *E*. *histolytica* antigen was analyzed by ELISA, Western immunoblot and dot blot analysis. In ELISA, all immune sera were reactive with C-Igl, rIgl-1, and rIgl-2. The reactivities of C-Igl were higher than those of rIgl-1 and rIgl-2 in all of the dilutions. The endpoint titers were more than 1:62,500 according to the cut-off value calculated by OD490 of control serum (Fig 1A). An increased level of C-Igl special IgG1 and IgG2 antibodies was detected in C-Igl-immunized sera. This demonstrated that IgG1 and IgG2 based immune responses exist in C-Igl induced immunity. Western immunoblot analysis showed that Igl antigens of *E*. *histolytica* HM-1:IMSS strain were recognized by pooled sera from C-Igl immune hamsters under reduced condition (Fig 1B). In the dot blot analysis using rIgl-1 and rIgl-2 antigen, pooled immune sera were reactive with both recombinant antigens, indicating that the immunization of hamsters by the C-terminal fragment of Igl-1 elicited antibodies reactive with both Igls (Fig 1C).

**Fig 2:**
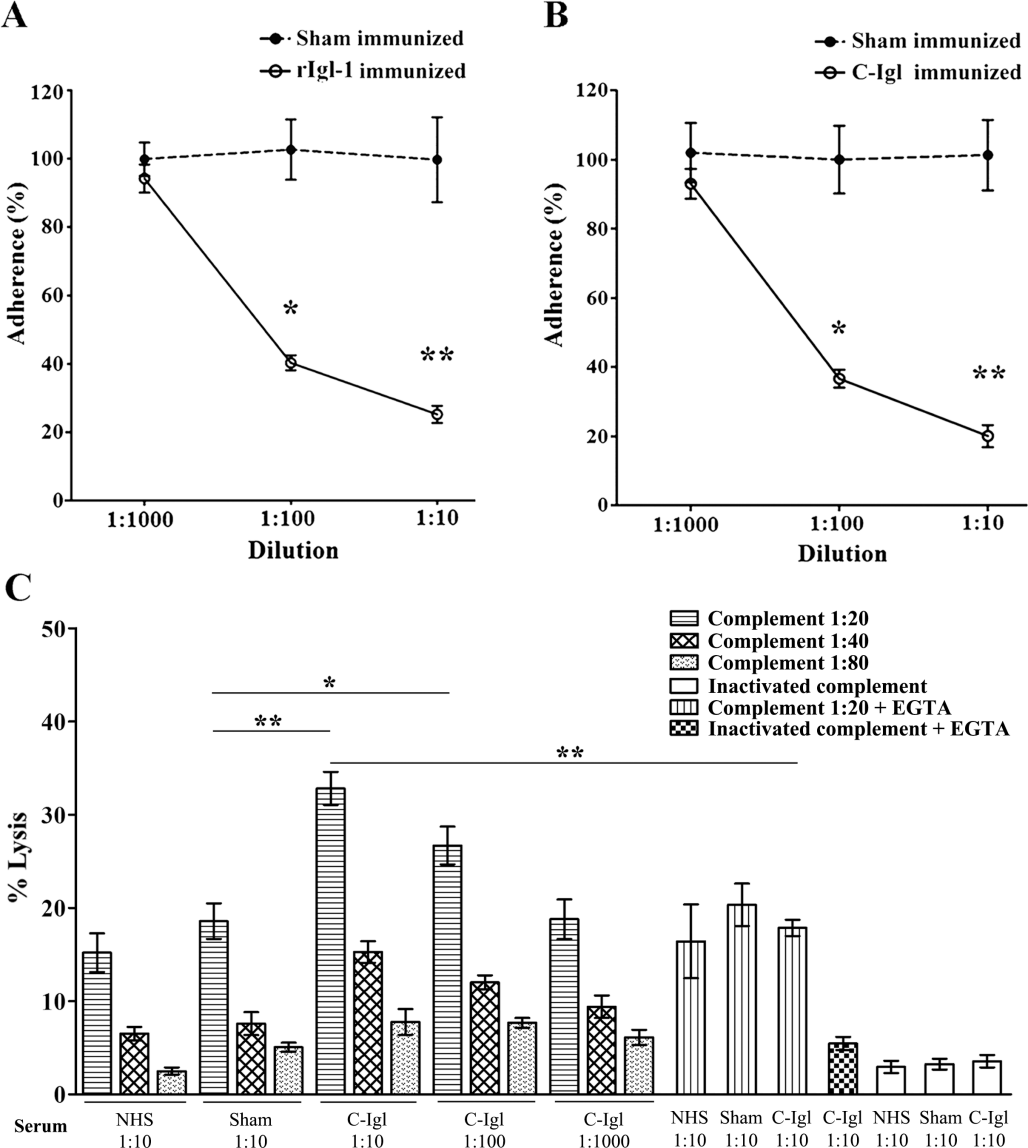
Effects of sera from hamsters vaccinated with the rIgl-1 (A) and C-Igl (B) on the adherence of CHO cells to *E*. *histolytica*. Trophozoites were pretreated with 1:1,000, 1:100, 1:10 diluted pooled sera from immunized hamsters and were then incubated with CHO cells. The number of trophozoites with at least three adherent CHO cells was scored by examining at least 300 trophozoites. Adherence was expressed as the percentage of adherence seen in phosphate-buffered saline -treated controls. Error bars represent the standard deviations of the means calculated from three independent replicates. *P < 0.001; **P < 0.001. (C) Complement-mediated lysis of E. histolytica by the sera from hamsters vaccinated with C-Igl. Trophozoites were incubated with different dilutions of serum from C-Igl immunized or sham-immunized hamsters and different dilutions of guinea pigs’ complement or heat-inactivated hamster serum (without complement). EGTA, which inhibits the classical complement cascade, served as additional controls for specificity. Cell lysis was measured 60 min after incubation. Heat-inactivated serum was used as a control. Error bars represent the standard deviations of the means calculated from four independent replicates. *P < 0.05; **P < 0.001 (vs. sham-immune control).

*E*. *histolytica* trophozoites were pretreated with pooled sera from the hamsters immunized with rIgl-1 or C-Igl at dilutions of 1:10, 1:100, and 1:1,000. After incubation with the CHO cells, the ability of trophozoites to adhere to the CHO cells was determined. As shown in Fig 2A and 2B, the adherence rate significantly decreased in comparison to preincubation with the sham-immunized sera. These data indicated that both of the antibodies to rIgl-1 and C-Igl recognized Igl of trophozoites and interfered with the ability of the parasite to bind to the CHO cells.

To examine whether antibodies from the hamsters immunized with C-Igl were able to exterminate trophozoites by complement-mediated lysis, the trophozoites were incubated with heat-inactivated serum from the immunized hamsters and guinea pigs’ complement. The numbers of living trophozoites significantly decreased after incubation with heat-inactivated C-Igl-immunized serum at 1:10 and 1:100 dilutions in comparison with control group in the presence of guinea pigs’ complement. This effect was also dependent on complement dose, and might be due to specific activation of the classical complement cascade, for trophozoites lysis was abolished by presence of 2mM Mg^2+^ and 10mM EGTA. Trophozoites lysis proceeded in the sham-immunized control group indicated the presence of antibody-independent activation of the alternative complement pathway. Spontaneous trophozoites lysis in the assays was minimal (Fig 2C). These data indicated that immunization with C-Igl fragments stimulated antibodies that might accelerate amebic lysis by activation of the complement.

### Expression of cytokine genes in immune hamsters

**Fig 3:**
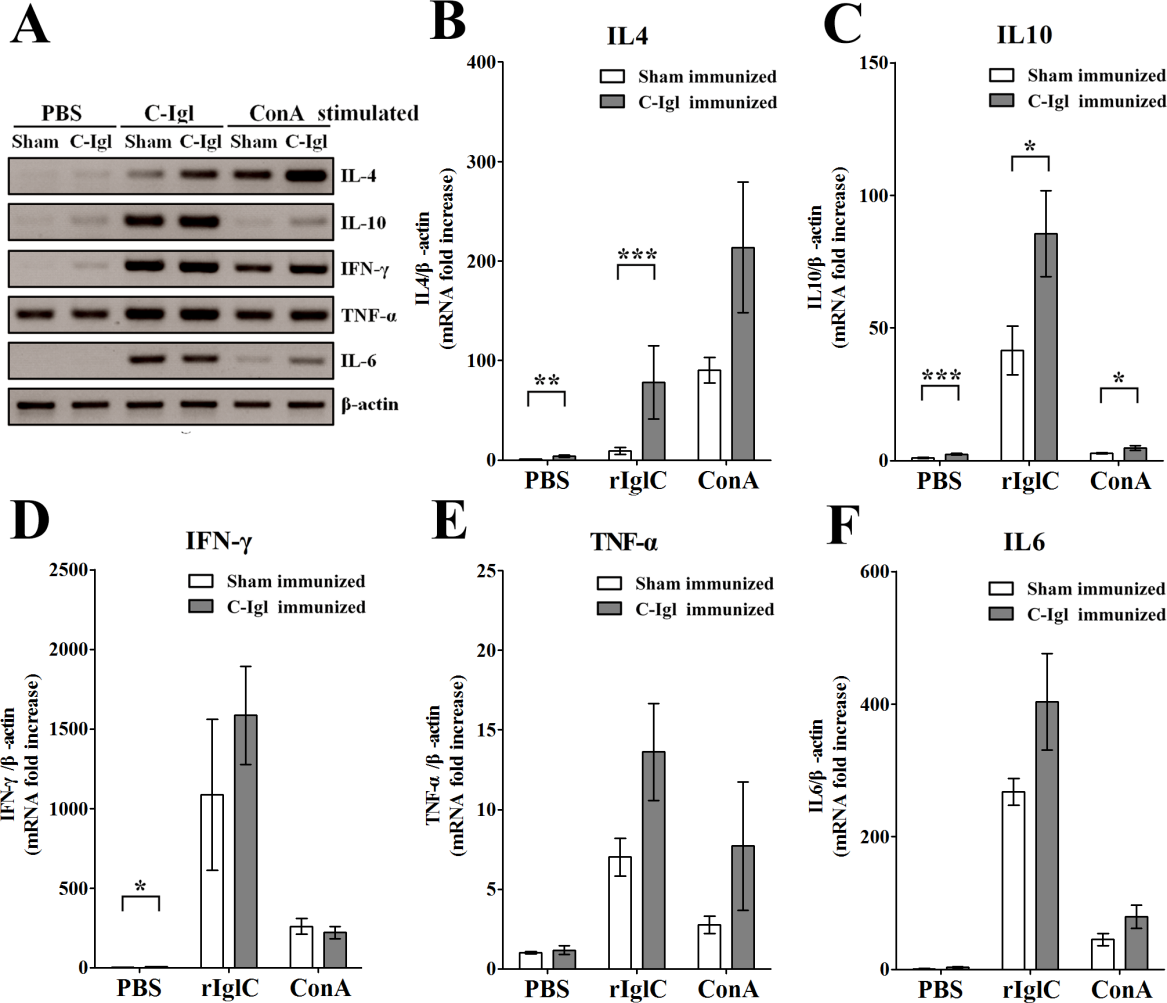
One-step RT-PCR amplification of cytokine genes in spleen cells from hamsters immunized with C-Igl (A) and quantitative real-time PCR assays of cytokine response induced by C-Igl in spleen cells from hamsters immunized with C-Igl (B-F). (A) The RT-PCR products were analyzed by electrophoresis. IL-4, IL-10, IFN-γ, TNF-α, IL-6, and β-actin data were included. (B-F) The expression level of cytokine genes of spleen cells from immunized hamsters was represented by the 2^-ΔΔCt^ of target cytokine gene relative to the β-actin gene and normalized with the corresponding values of sham-immunized PBS-stimulated group. White bars represent sham-immunized group data and gray bars represent C-Igl immunized group data. The Y axes were linear coordinates and numbers corresponded to the fold increase over value 1.0 given to the sham-immunized PBS stimulated control group. Error bars represent the standard errors of the means calculated from three independent replicates. Abscissa means different stimulates. *P < 0.05; **P < 0.01; ***P < 0.001.

We investigated the expression of Th2-type and Th1-type cytokines in the protection elicited by Igl. Spleen cells from the sham-immunized or C-Igl-immunized hamsters were stimulated by PBS, C-Igl, or ConA for 48 h *in vitro*. After restimulation *in vitro* with C-Igl, RNA extracted from spleen cells from the immunized hamsters was analyzed by one-step RT-PCR using primers specific for cytokine genes. Compared with those in the sham-immunized hamsters, the expression level of IL-4 and IL-10, but not of IFN-γ, TNF-α, or IL-6 were increased in the immunized hamsters (Fig 3A).

The technique of qRT-PCR was used to confirm the expression patterns of cytokine genes in the spleen cells of the hamsters immunized with C-Igl or PBS using the same primers as in the one-step RT-PCR assay. After *in vitro* stimulation with C-Igl, the expression of IL-4 gene was significantly elevated (*P* < 0.001) in the C-Igl-vaccinated hamsters with an 8.4-fold increase compared with that in the sham-immunized group (Fig 3B). The expression of IL-10 gene was also significantly higher by approximately two-fold (*P* < 0.001) in the vaccinated group compared with that in the control group (Fig 3C). These results indicated that the mRNA levels of IL-4 and IL-10 were clearly upregulated in the vaccinated hamster group. However, the production of the Th1 cytokine IFN-γ, pro-inflammatory cytokine TNF-α, and IL-6 in the C-Igl- vaccinated spleen cells was comparable with that in the control cells (Fig 3D–3F).

### Distinctive expression of PD1 and PD1 ligands' genes in C-Igl immunized hamsters

**Fig 4:**
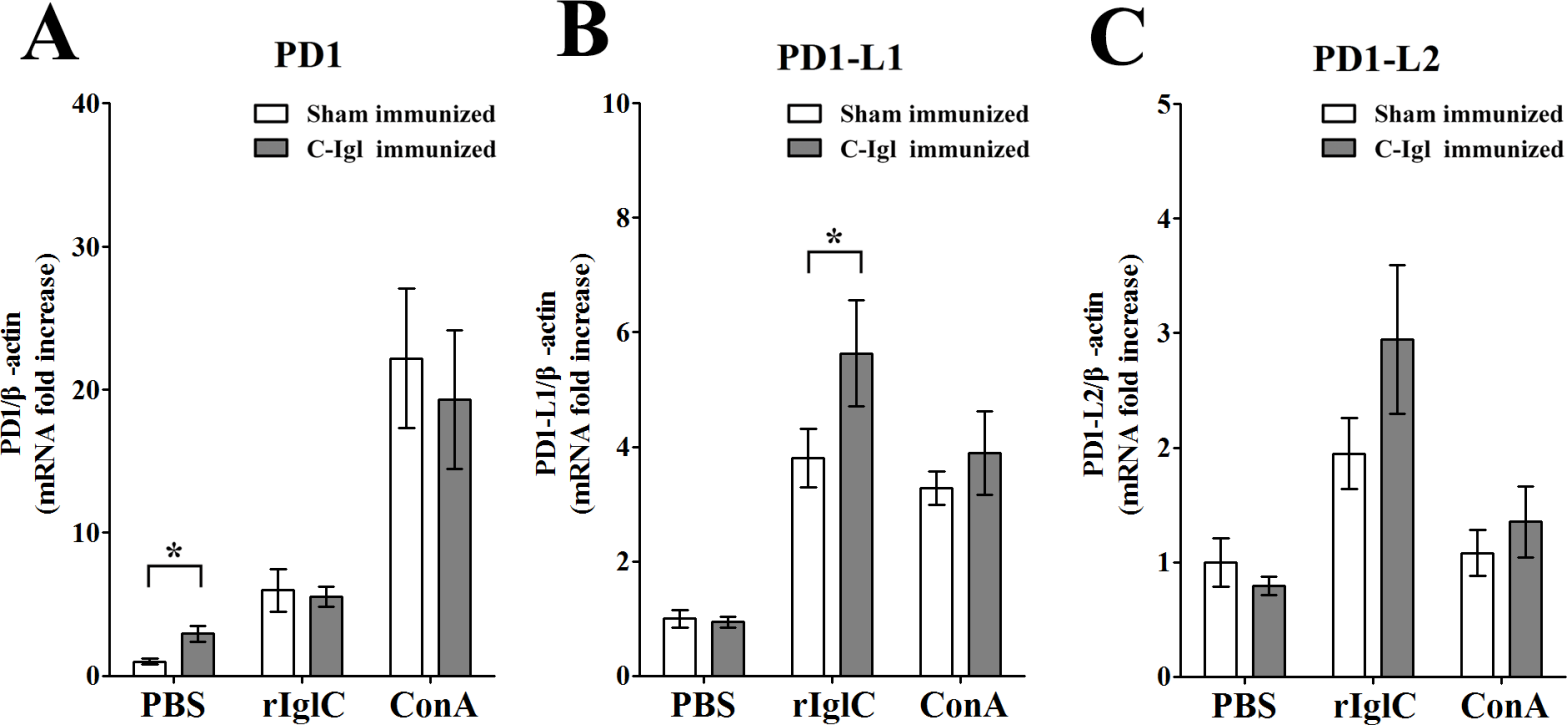
Quantitative real-time PCR assays of PD1 (A), PD1-L1 (B) and PD1-L2 (C) genes induced by C-Igl in spleen cells from hamsters immunized with C-Igl. The expression level of cytokine genes of spleen cells from immunized hamsters was represented by the 2^-ΔΔCt^ of target gene relative to the β-actin gene and normalized with the corresponding values of sham-immunized PBS-stimulated group. White bars represent sham-immunized group data and gray bars represent C-Igl immunized group data. The Y axes were linear coordinates and numbers corresponded to the fold increase over value 1.0 given to the sham-immunized PBS stimulated control group. Error bars represent the standard errors of the means calculated from three independent replicates. Abscissa means different stimulates. *P < 0.05.

qRT-PCR was used to confirm the expression of PD1 and PD1 ligands’ genes in the spleen cells after vaccination. *In vitro* stimulation with PBS resulted in higher PD1 expression (*P* < 0.05) in the vaccinated group compared to the control group (Fig 4A). In addition, PD1-L1 production was significantly upregulated in C-Igl-vaccinated hamsters (*P* < 0.05) with an 1.5-fold increase after C-Igl stimulation (Fig 4B**)**. On the other hand, the amount of PD1-L2 was not significantly changed in stimulated groups (Fig 4C).

## Discussion

Patients who recover from amebiasis acquire a certain level of resistance to *E*. *histolytica* reinfection [38, 39]. Effective vaccines to prevent amebiasis could enable the eradication of *E*. *histolytica*. Vaccines targeting native and recombinant forms of the protozoan Gal/GalNAc lectin proteins have been successful in protecting animals against intestinal amebiasis and ALA [18, 40–43]. In a previous study, we demonstrated that vaccination with native Igl can protect hamsters from ALA formation [26]. However, we could not completely rule out the possibility of contamination with other proteins in the fraction. In addition, this fraction appeared to contain both Igl-1 and Igl-2. The current study demonstrated that vaccination with rIgl-1 prepared in bacteria was effective for the prevention of ALA formation. Although we did not evaluate the efficacy of Igl-2 as a vaccine, Igl-1 could be a suitable vaccine candidate because the Western immunoblot analysis and dot blot analysis suggested that antibodies to Igl-2 were also elicited by immunization with Igl-1.

The most important observation in the current study is the effective protection achieved with C-Igl vaccination, suggesting that the domain responsible for protection from ALA formation was located in C-Igl. The result of the current study was also consistent with a previous observation, in which C-Igl is strongly recognized by sera from patients with amebiasis, including asymptomatic cyst passers [30].

The serum antibodies produced in response to C-Igl vaccination inhibited CHO cell adherence to trophozoites in a dose-dependent manner. Antibodies elicited by immunization with C-Igl demonstrated a complement-mediated cytolytic effect on trophozoites, probably due to specific activation of the classical complement cascade. These results established recombinant Igl as a candidate antigen for *E*. *histolytica* vaccines, which may be widely applied for protection against human amebiasis. It is known that an epitope on Hgl confer antibody-mediated protection against invasive amebiasis [43]. In Hgl, a carbohydrate recognition domain exists in the C-terminal region of the subunit. Although carbohydrate recognition domain has not yet been identified in Igl, a similar protective mechanism by antibodies may exist.

Extraintestinal abscesses, especially ALA, are a fatal feature of infection with *E*. *histolytica*. An important question is how this parasite adheres to and crosses the liver endothelium and induces abscess formation. Furthermore, the mechanisms underlying the protection of hamsters against ALA conferred by the intramuscular administration of C-Igl were not completely understood. The intramuscular route of immunization demonstrated a high efficiency in inducing a systemic protective response similar to the intraperitoneal immunization route. We hypothesized that after immunization, the C-Igl antigen could directly affect dendritic cells and that the activation of B cells induced systemic immune responses via antibodies. Thus, the intramuscular route showed classic advantages such as manageability, minimal invasiveness, and no requirement of special materials. Moreover, our results suggest that vaccination could yield antibodies to protect against the development of liver abscesses if the trophozoites manage to pass from the intestine to the liver and then adhere to and cross the liver endothelium.

Previous studies have shown that multivalent vaccines, such as Gal/GalNAc specific lectin, cysteine proteinase, serine-rich protein, and trophozoite surface metalloprotease EhMSP-1 [18, 42, 44], can indeed be developed. Successful delivery and expression of the recombinant C-Igl fragment in the prokaryotic expression system further indicate that it can be a basic component of an effective vaccine against human amebiasis.

As previously reported, once amebic infection is activated, T cells may participate in the immune response against *E*. *histolytica* through the following three pathways: (i) directly lysing trophozoites in a contact-dependent manner; (ii) producing cytokines, which activate effector cells for anti-ameba activity; and (iii) providing help for B-cell antibody production. A recent study provided evidence of amebicidal activity by both CD4^+^ and CD8^+^ T cells and transfer of protection conferred by Gal/GalNAc lectin-derived vaccines to naive animals merely by the transfer of immune T cells (CD4, CD8) [11]. In the current study, the C-Igl was identified as an effector molecule sufficient for immunizing hamsters. In addition, we demonstrated that a 48 h incubation of hamster spleen cells with C-Igl or ConA resulted in Th2 cytokine secretion and that this may contribute to Igl-mediated protection. The data also showed a correlation between partial and sterile protection against ALA with the local production of high levels of IL-4 and IL-10 genes by qRT-PCR.

A comparable level of IL-2 and IL-4 secreted by lymphoid cells from both spleen and hepatic lymph node of gerbils during the early stage of ALA development after responding to soluble amebic antigen. This finding indicated both Th1 and Th2 response phenotypes were present during amebiasis [45]. In general, the Th2 cytokine responses are characterized by IL-4, -5, -6, -9 and -10 productions. Th1 responses tend to promote cell-mediated immunity whereas Th2 responses are associated with humoral immunity. In our experiments, IL-4 and IL-10 were predominantly secreted by Th2 cells after vaccination with C-Igl. Given that B cells required assistance from T cells to complete their differentiation. IL-4 may function in amebic infections by facilitating antibody production of B cells when naive B cells circulate in the blood and lymph, pass through the secondary lymphoid organs, most notably to the spleen and lymph nodes. Thus, amebic antigen-specific T cells must act to promote antibody production, probably through cytokine release and contact-dependent stimulation of B cells.

Levels of a Th1 cytokine, IFN-γ, were evaluated in immunized hamsters and sham-immunized animals. The animals immunized with C-Igl and PBS presented an average of a 1.5-fold level difference that was not statistically significant (Fig 3D). Similarly, no statistical difference was found in TNF-α and IL-6 levels between the immunized hamsters and sham-immunized animals. Th2 clones are known to be involved in the production of IL-4 and IL-5 in addition to IL-6, IL-9, and IL-10, these cells play critical roles in generating humoral immune responses including the production of IgG1. Cytokines from Th2 cells can inhibit the actions of Th1 and vice versa. The regulation of balance of cytokine profile may be determined by the character of the certain antigens.

Although the expression levels of IFN-γ, TNF-α, and IL-6 in the C-Igl- and sham-immunized animal groups were similar after C-Igl stimulation *in vitro*, significant cytokine upregulation was observed between the PBS- and C-Igl-stimulated groups. One hypothesis is that C-Igl possesses the ability to activate Th1 and pro-inflammatory cytokines. To verify this hypothesis, spleen cells from the non-immunized hamsters and mice were used in *in vitro* stimulation assays. Similar RNA expression levels of cytokines by spleen cells were observed in the non-immunized hamsters (S1A Fig) compared with those in the sham-immunized hamsters (see Fig 3). The results indicated that expression levels of IFN-γ, TNF-α, and IL-6, but not IL-4 were elevated in the C-Igl-stimulated hamster and mouse groups (S1A and S1B Fig), both hamster and mouse spleen cells were incapable of proliferation after C-Igl stimulation (S1C and S1D Fig). In comparison, IL-4 level was elevated in the C-Igl-immunized hamsters when the cells were activated by C-Igl (see Fig 3B). These data indicated that IL-4 secretion was an antigen specific reaction to the vaccine and IL-4 may play an important role in protecting hamsters from ALA in C-Igl-mediated protection.

Twenty-four hours after trophozoites inoculation to the liver, macrophages at the periphery of the lesions begin to form an ill-defined border between the parenchymal cells and central necrotic area [46]. In addition, IL-4 has been reported to play a pathogenic role in the persistence of *E*. *histolytica* infection through the suppression of protective IFN-γ in intestinal infection [47]. After vaccination with C-Igl, IL-4 and IL-10 were predominantly secreted by Th2 cells. Both of these cytokines may elicit amebicidal action by suppressing macrophages in the liver, as well as controlling excessive inflammation and protecting hamsters from ALA formation. In intestinal and extraintestinal amebiasis, host inflammatory response contributes to tissue damage. TNF-α was a critical cytokine mediator of tissue destruction during amebiasis [48] and the anti-inflammatory cytokine IL-10 is an important immunoregulator [49]. IL-10 counteracts an exaggerated pro-inflammatory immune response by inhibiting the production of inflammatory mediators such as TNF-α.

The PD1–PD1-L pathway, including the PD1 receptor and its two ligands, PD1-L1 and PD1-L2, could regulate the activation, inhibition and fine-tuning of T-cell responses. Moreover, PD1-L1 was elevated after *in vitro* stimulation with C-Igl in C-Igl-vaccinated hamsters. The PD1–PD-L pathway is regarded as a key regulator of the crucial balance signals needed for effective immune responses [50] and main avoidance of an excessive immune response in the presence of foreign antigens [51] that could exert synergistic effects with IL-10. It has also been reported that expression of PD1-L1 is correlated with secretion of IL4 [52]. IL-4 supports the generation of Th2 cells, which simultaneously activate B cells to induce systemic immune responses and yield antibodies to protect against the development of liver abscesses. Previously, longitudinal field studies also demonstrated an association between anti-lectin antibody, IL-4, and immunization of ALA [44, 53]. Not coincidentally, in an analysis of the immune response to hepatitis B virus infection, it was found that patients who recovered from acute hepatitis B virus infection activated the Th2 release of IL-4 and IL-10, which induced the B-cell production of antibodies [54]. Thus, the results presented here demonstrated a model in which C-Igl induces Th2 response, which then generates a cell-mediated immune response. Possibly through IL-4 and IL-10 produced by antigen-specific Th2 cells mediated immunity and collaborated with B-cells for antibody production [55].

In summary, the results presented in the current study provide support for the further development of C-Igl as a target antigen for vaccination to prevent invasive amebiasis. The results also provide new insights into Igl, a cysteine-rich surface antigen of *E*. *histolytica*. Further studies are underway to explore preliminary steps toward the development of a human amebiasis vaccine.

## Supporting Information

S1 Fig Spleen cells stimulation assay in non-immunized hamsters and mice. *Gene expression levels of cytokines in non-immunized hamster spleen cells (A)*. Gene expression levels of cytokines in non-immunized hamster spleen cells were represented by the 2^−ΔΔCt^ method with transcripts of the β-actin gene used as the internal reference control. The white, gray, and black bars denote data for the PBS-, C-Igl-, and ConA-stimulated groups, respectively. The Y axis were linear coordinates and numbers correspond to fold increase over value 1.0 given to the PBS-stimulated control group. Error bars represent the standard errors of the means calculated from three independent replicates. *The protein levels of cytokines in non-immunized mice spleen cells (B)*. The protein levels of cytokines (pg/ml) in non-immunized mice spleen cells in the culture supernatant were determined by ELISA. The white, gray, and black bars denote data for the PBS-, C-Igl-, and ConA-stimulated groups, respectively. Error bars represent the standard errors of the means calculated from three independent replicates. *Cell proliferation assay of non-immunized hamster spleen cells (C)*. Cell proliferation was measured by using the CCK-8 kit after PBS, C-Igl, or ConA stimulation. The white and black bars denote data for 24 h and 48 h stimulation groups. The Y-axis shows the optical density (OD) values of the test well minus the blank well. Error bars represent the standard errors of the means calculated from four independent replicates. *Cell proliferation assay of non-immunized mice spleen cells (D)*. Cell proliferation was measured by using the CCK-8 kit after PBS, C-Igl, or ConA stimulation. The white and black bars denote data for 24 h and 48 h stimulation groups. The Y-axis shows the optical density (OD) values of the test well minus the blank well. Error bars represent the standard errors of the means calculated from four independent replicates.
(TIF)

S1 Text Analysis of cytokine protein levels in mice and spleen cell proliferation assay. (PDF)
