# Supplementary figures and images for: Evaluation of the C-Terminal Fragment of Entamoeba histolytica Gal/GalNAc Lectin Intermediate Subunit as a Vaccine Candidate against Amebic Liver Abscess
Source: PLoS Negl Trop Dis. 2016 Jan 29;10(1):e0004419. doi: 10.1371/journal.pntd.0004419 (PMC4732598; doi:10.1371/journal.pntd.0004419)

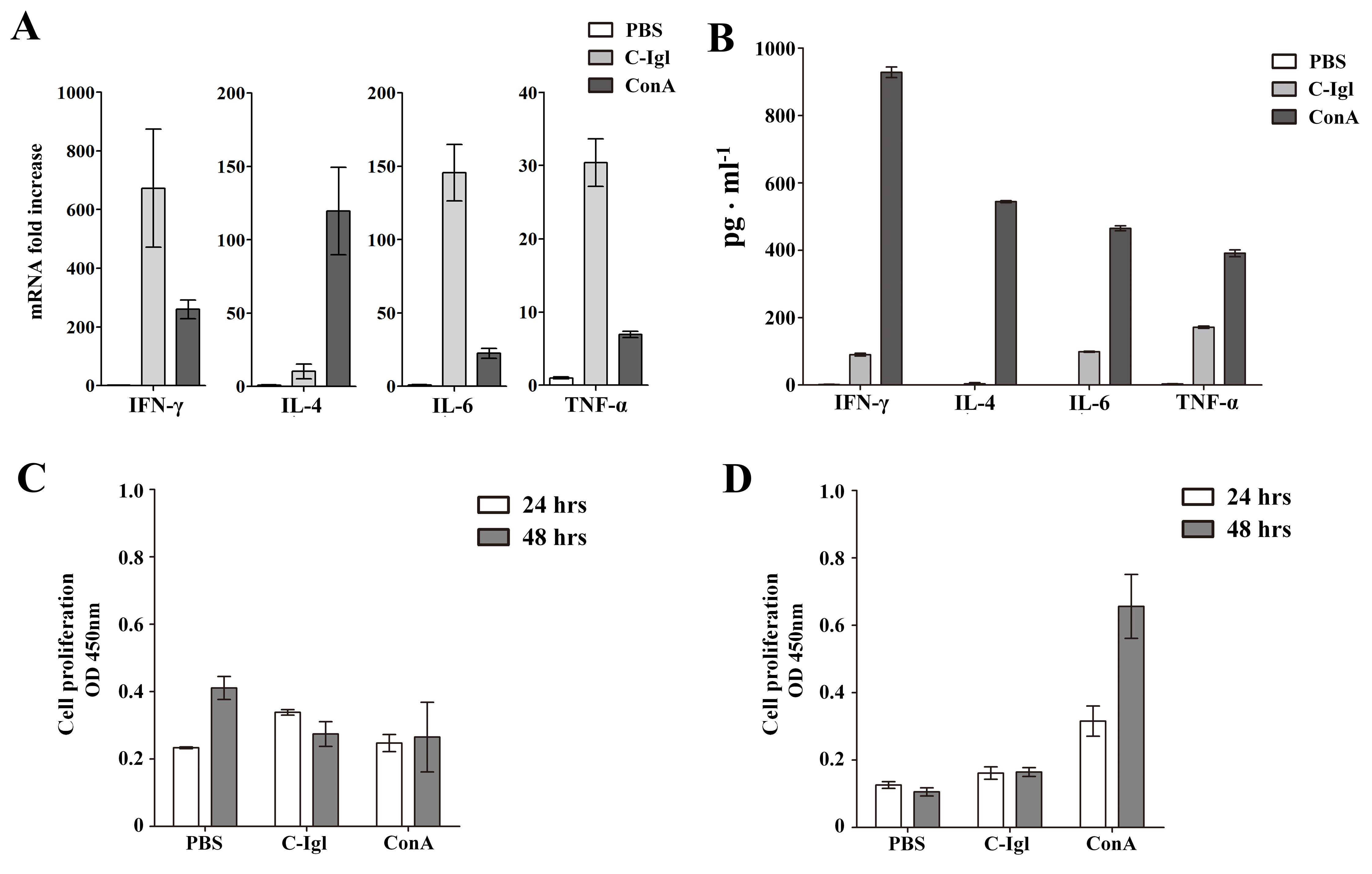

Supplement: S1 Fig — Gene expression levels of cytokines in non-immunized hamster spleen cells (A). Gene expression levels of cytokines in non-immunized hamster spleen cells were represented by the 2−ΔΔCt method with transcripts of the β-actin gene used as the internal reference control. The white, gray, and black bars denote data for the PBS-, C-Igl-, and ConA-stimulated groups, respectively. The Y axis were linear coordinates and numbers correspond to fold increase over value 1.0 given to the PBS-stimulated control group. Error bars represent the standard errors of the means calculated from three independent replicates. The protein levels of cytokines in non-immunized mice spleen cells (B). The protein levels of cytokines (pg/ml) in non-immunized mice spleen cells in the culture supernatant were determined by ELISA. The white, gray, and black bars denote data for the PBS-, C-Igl-, and ConA-stimulated groups, respectively. Error bars represent the standard errors of the means calculated from three independent replicates. Cell proliferation assay of non-immunized hamster spleen cells (C). Cell proliferation was measured by using the CCK-8 kit after PBS, C-Igl, or ConA stimulation. The white and black bars denote data for 24 h and 48 h stimulation groups. The Y-axis shows the optical density (OD) values of the test well minus the blank well. Error bars represent the standard errors of the means calculated from four independent replicates. Cell proliferation assay of non-immunized mice spleen cells (D). Cell proliferation was measured by using the CCK-8 kit after PBS, C-Igl, or ConA stimulation. The white and black bars denote data for 24 h and 48 h stimulation groups. The Y-axis shows the optical density (OD) values of the test well minus the blank well. Error bars represent the standard errors of the means calculated from four independent replicates. (TIF) [file pntd.0004419.s001.tif]
