# Supplementary material for: Evaluation of the C-Terminal Fragment of Entamoeba histolytica Gal/GalNAc Lectin Intermediate Subunit as a Vaccine Candidate against Amebic Liver Abscess
Source: PLoS Negl Trop Dis. 2016 Jan 29;10(1):e0004419. doi: 10.1371/journal.pntd.0004419 (PMC4732598; doi:10.1371/journal.pntd.0004419)
Supplement: S1 Text — (PDF) [file pntd.0004419.s002.pdf]

**Analysis of cytokine protein levels in mice by enzyme-linked immunosorbent assay.** To assess whether C-Igl possessed the ability to activate Th1 cytokines and proinflammatory cytokines, spleens from non-immunized mice were harvested and processed for cell suspensions after red blood cell lysis using the 0.84% $NH_4Cl$ buffer. Spleen cells ($2 \times 10^6$) were cultured at 37 °C in 5% $CO_2$ and in 24-well culture plates (Costar) containing RPMI 1640 medium (Invitrogen) supplemented with 10% fetal bovine serum, 100 U/ml penicillin, and 100 μg/ml streptomycin. Cells were stimulated with PBS, 5 μg/ml C-Igl protein or 5μg/ml ConA. After 48 h of incubation, the supernatants were collected and analyzed using a Mouse CK ELISA kit (Quantikine, R&D, USA), which included the following cytokines: IFN-γ, TNF-α, IL-4, and IL-6. Samples were processed following the manufacturer's instructions and measured in pg/ml of supernatant.

**Spleen-cell proliferation assay.** In the cell proliferation assay, spleens from non-immunized hamsters or mice were harvested and processed for cell suspensions. Spleen cells ($2 \times 10^5$) were cultured at 37 °C in 5% $CO_2$ and in 96-well culture plates (Costar) containing RPMI 1640 medium (Invitrogen) supplemented with 10% fetal bovine serum, 100 U/ml penicillin, and 100 μg/ml streptomycin. Cells were stimulated with PBS, 5 μg/ml C-Igl protein or 5 μg/ml ConA. CCK-8 reagents (DOJINDO, Japan) were added to each well at 20 h or 44 h, and optical density (OD) was measured at 450 nm using a microplate reader (Bio-rad, USA) at 24 h or 48 h.
